# Simple Extraction of Cannabinoids from Female Inflorescences of Hemp (*Cannabis sativa* L.)

**DOI:** 10.3390/molecules27185868

**Published:** 2022-09-10

**Authors:** Milena Szalata, Mariola Dreger, Aleksandra Zielińska, Joanna Banach, Marlena Szalata, Karolina Wielgus

**Affiliations:** 1 Department of Biotechnology, Institute of Natural Fibres and Medicinal Plants National Research Institute, Wojska Polskiego 71B, 60-630 Poznań, Poland; 2 Institute of Human Genetics, Polish Academy of Sciences, Strzeszyńska 32, 60-479 Poznań, Poland; 3 Department for Research and Processing of Seeds, Institute of Natural Fibres and Medicinal Plants National Research Institute, Wojska Polskiego 71B, 60-630 Poznań, Poland; 4 Department of Biochemistry and Biotechnology, Poznań University of Life Sciences, Dojazd 11, 60-632 Poznań, Poland; 5 Department of Pediatric Gastroenterology and Metabolic Diseases, Poznan University of Medical Sciences, Szpitalna Street 27/33, 60-572 Poznań, Poland

**Keywords:** *Cannabis sativa* L., Δ^9^-tetrahydrocannabinol (Δ^9^-THC), cannabidiol (CBD), water extraction, ethanol extraction

## Abstract

The high interest in non-psychoactive cannabidiol increases the need for efficient and straightforward cannabidiol (CBD) extraction methods. The research aimed to compare simple methods of cannabinoid extraction that do not require advanced laboratory equipment. This work assesses the content of total CBD and Δ^9^-tetrahydrocannabinol (Δ^9^-THC) in popular solvents such as water and ethanol extracts. Hemp raw material was analyzed with Gas Chromatography with a Flame Ionization Detector (GC-FID), while extracts were tested by High-Performance Liquid Chromatography (HPLC). The female inflorescences of three varieties of industrial hemp were tested: Futura 75, KC Dora, and Tygra (different sowing and N fertilization densities). Tygra (T/10/30) showed the highest content of CBD (0.064%) in water extracts. However, in 80% tincture from Futura 75 (F/30/30), a higher CBD content of 1.393% was observed. The use of 96% ethanol for extraction and ultrasound enabled the highest CBD content to be obtained: 2.682% in Futura 75 (F/30/30). Cold water extraction showed no effect on Δ^9^-THC content, while hot water extraction increased content from 0.001% in KC Dora to 0.002% in Futura 75 (F/30/30) and Tygra, but the changes were statistically insignificant. Application of 80% ethanol revealed the significantly highest content of Δ^9^-THC in KC Dora, from 0.026% (K/30/90) to 0.057% (K/30/30), as well as in Tygra (T/30/30) (0.036%) and Futura 75 (F/30/30) (0.048%). The use of ethanol extraction in combination with ultrasound could be an efficient method of obtaining cannabinoids.

## 1. Introduction

Hemp (*Cannabis sativa* L.) is an annual plant that belongs to the genus *Cannabis*. Monoecious forms with male and female flowers on the same plant and dioecious forms with male and female flowers on different plants can be distinguished [1]. Since ancient times, the most general application of *Cannabis sativa* has been its use as a medicine and a narcotic. Hemp has been cultivated for different purposes, including fiber, textiles, fuels, paper, building materials, food, essential oils, and cosmetics [2,3,4,5]. *Cannabis sativa* produces approximately 500 pharmacologically interesting compounds. The best known compounds with potential therapeutic activity are cannabinoids–unique hemp substances. Commonly known cannabinoids include Δ^9^-tetrahydrocannabinol (Δ^9^-THC, psychoactive) and cannabidiol (CBD, non-psychoactive). This group also includes Δ^8^-tetrahydrocannabinol (Δ^8^-THC), cannabigerol (CBG), and cannabinol (CBN). Cannabinoids are created by the plant from carboxylic acid: Δ^9^-tetrahydrocannabinolic acid (Δ^9^-THCA) and cannabidiolic acid (CBDA). Neutral cannabinoids are formed (decarboxylation process) during exposure to light, heat, or as a result of prolonged storage: Δ^9^-THC, CBD [3,6,7,8].

The condition permitting the cultivation of cannabis in the country is the level of Δ^9^-THC < 0.2%. The annual control of hemp cultivation in European Union countries allowed the creation of an annually updated list of cannabis varieties with a stable Δ^9^-tetrahydrocannabinol content below 0.3%. This list is available on the European Union website in the EU Plant variety database catalog (v.3.4) and the Common catalog of varieties of agricultural plant species [9].

Hemp easily adapts to changing growing conditions. The numerous types, forms, and varieties are biologically, morphologically, and economically diverse. Depending on the adaptation to environmental conditions, it is possible to distinguish northern hemp (usually below 80 cm) with a short growing period (60–75 days) with thick fiber, southern hemp (300–400 cm) with a long growing period (140–160 days) and a high yield of good quality fiber. The most adapted to the conditions in Poland are the Central European varieties (200–300 cm) with a relatively short growing season (80–120 days) [10].

The medicinal properties of cannabis-based preparations have long been known in medicine. However, the full use of the potential of cannabis-produced substances is limited by their narcotic nature [11,12]. Studies conducted in recent years indicate that cannabis, cannabinoids, terpenes, terpenoids, and flavonoids with medical potential have been identified. These compounds, mainly through the endocannabinoid system, can influence human physiological processes, the immune system, or inflammation, and numerous studies indicate the legitimacy of their use in medicine [13].

Hemp extraction is of great interest to the hemp industry. Cannabidiol (CBD) extracts are developed as medical marijuana, and whole plant tetrahydrocannabinol (THC) extracts for the recreational cannabis market ranging from e-cigarette oils to candy. It is assumed that both applications require more and more purity and meet specific parameters [14]. New technologies have enabled the implementation of new forms of a cannabis extract, such as oral tinctures, e-cigarette oils, and wax concentrates [15,16].

Fibrous hemp contains CBD with a low content of Δ^9^-THC (<0.3%) simultaneously. Therefore, it may have valuable healing properties. Cannabinoid content depends on biotic factors: the genotype of the plant, the development phase, and abiotic factors, including growth conditions, e.g., light, temperature, nutrients, or UV radiation. The highest concentration of cannabinoids occurs in inflorescences, then in leaves, and the lowest concentration is in stems [1,2,17]. Due to the high variability of cannabinoids, including THC content, hemp growers and the hemp industry are interested in changing the limits.

Hemp is not only used as a narcotic (recreational) but is popularly consumed for medicinal purposes. Legislative changes according to the legal use of cannabis as a therapeutic remedy in a few countries and U.S. states widened the method of administration, such as vaporization, edibles, and liquid tinctures as an alternative to smoking [18,19,20]. There are also numerous low-THC cannabis products available in Europe, including herbs, resin, oil, smoking products of e-liquids and crystals, and various edible cannabis products. The sale also includes lotions, creams, and pastes [21].

Cannabis has long been used to make preparations to: reduce nausea and vomiting during chemotherapy, improve appetite in HIV-infected people, or reduce muscle spasms. Cannabinoids, which are secondary metabolites, are responsible for the therapeutic effect, so there is a great demand for their production in plants [8]. Δ^9^-THC is a remedy for vomiting and nausea relief associated with cancer treatment, analgesia, and appetite stimulation in AIDS and anorexia patients with wasting syndrome. As a non-psychoactive compound, CBD also showed antibacterial and anti-inflammatory activity and immune-modulating properties. Δ^9^-THC excess in users may cause a panic attack, anxiety, and problems with heart and blood pressure [7,22].

The growing interest in non-psychoactive cannabidiol and its pharmacological potential has allowed for the development of efficient methods of cannabinoid extraction, including CBD, and their quantitative analysis both in plant material and in products prepared based on cannabis [23,24,25]. Currently, many CBD products are appearing on the market [26]. Unfortunately, the law prohibits the cultivation of narcotic cannabis, which includes a high content of both Δ^9^-THC and CBD (the CBD to Δ^9^-THC ratio is very close to 1:1). The legislation in Poland allows the cultivation of hemp with the Δ^9^-THC content below 0.2%. Still, the European Union Parliament recently raised the limit for industrial hemp of any THC to 0.3% within the EU’s standard agricultural policy (CAP). Such plants are characterized by their CBD content.

The choice of the extraction method is determined by the desire to preserve the aroma and taste based on terpenes or the purity of the product in the absence of solvents. Solvent-free methods are assumed to be simpler and more natural but have lower mass yields and concentration factors than other methods. Some countries also have legal regulations that use specific extraction methods [27]. Some extraction methods are relatively new and have been slightly modified in recent years [16,28,29]. Other methods are thousands of years old. In 2018, Kenneth Morrow distinguished eight main extraction methods and the 12 most popular forms of cannabis extracts [30]. The extraction methods include the following: dry-sieved then pressed hashish; water extracted, dried, and pressed hashish (mechanical separation); rosin, heat, and pressure applied, squeezing out the resin; liquid nitrogen extraction (butane, propane, hexene, etc.); carbon dioxide (CO_2_) extraction; ethanol alcohol extraction; hydrocarbon extraction; distillation, wiped film, thin-film or short path, optionally vegetable oils: coconut, olive oil, etc. In addition, among the primary forms of extracted cannabis, the following can be distinguished: pressed resin glands (hashish); oil form, sometimes referred to as RSO; tincture; shatter; budder; wax; high terpene full-spectrum extract (HTFSE); high cannabinoid full spectrum extract (HCFSE); vape cartridges; CBD in crystallized form; THCA in concentrated form; delta-8 in oil form.

Different extraction methods could extract cannabinoids from plant material, such as dynamic maceration (DM) and ultrasound-assisted extraction (UAE). The efficacy of the extraction technique depends on other parameters, including temperature, type of solvent (e.g., water, alcohol, or alcoholic solvents), plant material, and extraction time [31,32]. Applying traditional extraction methods, including water and ethanol extraction, might be an exciting solution. The research aimed to compare the most suitable simple cannabinoid extraction methods from fibrous plants and define whether hemp extracts could be a potential source of cannabinoids. The best-known extraction method uses a solvent with a high affinity for cannabinoids (ethanol, methanol, or hexane), while the water used to prepare the decoctions has a low affinity [33]. Nevertheless, it was decided to assess whether the water extracts could contain cannabinoids.

## 2. Results

### 2.1. Cannabinoids Content in Inflorescences

After drying, the collected inflorescences were analyzed for the content of cannabinoids. Due to the instability of the acid forms of cannabinoids and the increase in the number of neutral forms, the experiment assessed the total content of cannabinoids (including acid and neutral cannabinoids). Then the tested material was used to prepare the extracts and measure the total Δ^9^-THC and CBD content. The GC-FID analysis indicated that the selected varieties showed a low Δ^9^-THC content (below 0.2%). According to legal requirements, the amount of Δ^9^-tetrahydrocannabinol was significantly higher in the Polish variety Tygra (0.12% and 0.15%). In contrast, KC Dora contained 0.09% and 0.10% and Futura 75 contained 0.05 and 0.06% Δ^9^-THC respectively. The highest content of cannabidiol characterized the KC Dora variety at the level of 3.60% and 3.63%, respectively, in both sowing density and nitrogen dose combinations. In the Futura 75 variety (2.48% and 2.43%) and the Tygra variety (2.26% and 2.58%), CBD content was significantly lower (Table 1). Regardless of the sowing density and the dose of nitrogen fertilization, the analysis did not exhibit any significant differences in the content of cannabinoids within the variety. Significant differences in the amount of CBD and Δ^9^-THC were observed only between the varieties.

### 2.2. Extraction Methods

Extracting organic solvents is the most convenient method of obtaining medicinal cannabis. However, the need to use new approaches should be accepted, emphasizing the development of a green and sustainable cannabis extraction method [34]. Methods based on alcohol, butane, CO_2_, water, and ethanol commonly extract cannabinoids from plants. The hemp industry relies on the three most popular extraction techniques: alcohol extraction, hydrocarbon extraction, and supercritical CO_2_ extraction [14]. Each extraction method has advantages and disadvantages. The technique will determine the scale and the quality of cannabinoids, terpenes, and flavonoids. It is assumed that solvent-based extraction methods are used for large-scale extraction.

The hemp industry has encouraged people to use creative ways to extract cannabinoids from the cannabis plant [16,29,35]. However, a few methods of cannabis extraction are most widely used in the industry: ethanol extraction, alcohol tincture extraction, and supercritical CO_2_ extraction. Additionally, hydrocarbons are commonly used as a solvent. On a smaller scale, methods based, for example, on a professional rosin press can be used.

#### 2.2.1. Water Extraction

Water extraction uses a solvent-free method that immerses the cannabis plant in freezing water. The plant is stirred in cold water, which causes the hairs to separate from the plant. As the trichomes fall off the plant, they pass through screens. The result is a product with 50% to 70% THC levels. An in situ decarboxylation system (149.9 °C, 42 min) with pressurized hot water extraction (PHWE) was also developed, which made it possible to obtain cannabis extracts with high CBD content while reducing the content of THC and CBN [36]. The influence of cold and hot water on the cannabinoid content in extracts obtained from the tested plant material was assessed (Table 2).

High-performance liquid chromatography analysis revealed that a low level of CBD content characterized the cold water extraction of hemp inflorescences through 24 h from 0.005% in the Polish variety Tygra to 0.009% in the Hungarian hemp KC Dora. Additionally, in all types, Δ^9^-THC was not detected. The effect of different agricultural conditions was not observed. Application of temperature (3 min boiling) increased cannabidiol extraction significantly. The highest content of CBD was found in the Futura 75 (F/10/90) and Tygra (T/10/30) varieties at 0.063% and 0.065%, respectively. Slightly lower content of CBD was found in KC Dora (K/30/30), 0.054%. In the hot water extract, the impact of sowing density and nitrogen dose was observed, because significantly lower content of cannabidiol was observed in the varieties Futura (F/30/30) (0.048%), Tygra (T/30/30) (0.049%), and KC Dora (K/30/90) (0.051%). Hot water extraction revealed the appearance of an insignificantly low amount of Δ^9^-THC at the level of 0.001% in the variety KC Dora (K/30/30 and K30/90) to 0.002% in Futura 75 (F/30/30) and Tygra (T/10/30 and T/30/30).

#### 2.2.2. Ethanol Extraction

Methods of cannabinoid extraction include extraction using ethanol as a solvent. Due to its polar nature, ethanol is a suitable solvent for extracting cannabinoids.

**Figure 1:**
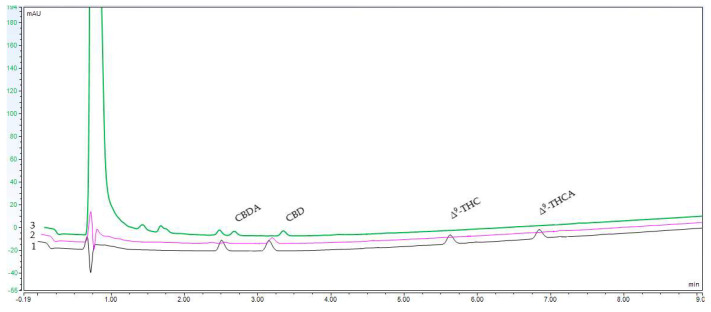
Representative HPLC chromatograms of (**1**) reference compounds, (**2**) water extract, and (**3**) ethanol extract.

Representative HPLC chromatograms of reference compounds and water and ethanol extracts are shown in Figure 1.

For the experiment, three ethanol concentrations were used in combination with dynamic maceration (Table 3).

Analysis of 20%, 40%, and 80% tinctures revealed that the significantly lowest content of CBD was obtained by extraction with 20% ethanol in all tested varieties at the level from 0.063% in Tygra (T/30/30) to 0.081% in Futura 75 (F/10/90), while the content of CBD was at the same level as in the hot water extract. On the other hand, the Δ^9^-THC level was not found to be similar to the extraction with cold water. However, unlike the water extracts, ethanol seems to allow more stable and repeatable sections to be obtained. Analysis of 40% tinctures showed significantly better extraction of CBD. In the Tygra variety, the content of cannabidiol was at the level of 0.535% (T/10/30) and 0.557% (T/30/30); in the Futura 75 variety, the analysis showed a slightly higher content of CBD from 0.581% (F/10/90) to 0.616% (F/303/30); and the highest amount of CBD was found in 40% tincture from the variety KC Dora at the level of 0.616% (K/30/30) and 0.619% (K/30/90). The differences were insignificant despite the variation in the cannabinoid content in 40% tinctures of the three varieties.

The presence of Δ^9^-THC was found in 40% tinctures at the level of 0.001% in the variety KC Dora (K/30/30) and Tygra at the level from 0.002% (T/10/30) to 0.003% (T/30/30) and in the variety Futura 75 at 0.004% (F/10/90). The analysis of Δ^9^-THC content showed the impact of sowing density and nitrogen dose in the Futura 75. A significantly higher range of Δ^9^-THC was observed at 0.006% in the Futura 75 variety (F/30/30) harvested from the plot with a sowing density of 30 kg⋅ha^−1^ and nitrogen dose of 30 kg⋅ha^−1^.

In 80% tinctures, the content of cannabinoids was significantly higher than in 20% and 40% tinctures. Additionally, the impact of different agricultural conditions on the range of cannabinoids within varieties was also observed. In 80% tinctures, the significantly lowest content of CBD was found in the Tygra variety at the level of 0.952% (T/30/30) and 0.997% (T/10/30). The differences within the variety were also significant. A similar effect was observed in Δ^9^-THC content at the level of 0.036% (T/30/30) and 0.045% (T/10/30), and these differences are also statistically significant. In the KC Dora variety, the content of cannabinoids was significantly higher than in the previous extract, and the differences within the array are also substantial. The content of CBD was at the level of 1.262% (K/30/30) and 1.169% (K/30/90), and the Δ^9^-THC content was at the level of 0.057% and 0.026%, respectively. The analysis of 80% tincture obtained from the Futura 75 variety showed the highest content of CBD from 1.305% (F/10/90) to 1.393% (F/30/30), and the Δ^9^-THC content at the level of 0.054% (F/10/90) and 0.048% (F/30/30).

Ultrasound extraction with 96% ethanol revealed a similar tendency in CBD content in the tested hemp varieties (Table 4). A higher concentration of ethanol in combination with ultrasound allowed us to obtain extracts containing CBD at a level from 1.945% in the Tygra (T/10/30) to 2.682% CBD in the Futura 75 (F/30/30) variety. There was also a tendency to obtain the highest CBD in the extracts from the Futura 75 variety. A slightly lower content of cannabidiol was found in the KC Dora variety and the weakest in the Tygra variety.

The analysis of 96% ethanol extracts showed the presence of Δ^9^-THC content ranging from 0.022% (K/3090) and 0.029% (K/30/30) in an extract from the variety KC Dora to 0.035% (T/30/30) and 0.045% (T/10/30) in the variety Tygra and 0.039% (F/10/90) and 0.057% (F/30/30) in the variety Futura 75.

Analysis of the variety Futura 75 during all extraction stages revealed a significant increase in CBD content in 80% extracts to 1.305% (F/10/90) and 1.393% (F/30/30). In cold water extract, the content of CBD was 0.007% (F30/30), and it ranged from 0.0048% (F/30/30) to 0.063% (F/10/90) in hot water extract. On the other hand, in 20% ethanol extracts it ranged from 0.071% (F/30/30) and 0.081% (F/10/90), while in 40% ethanol extracts it reached 0.581% (F/10/90) and 0.616% (F/30/30) (Table 5). The highest content of CBD was observed in 96% extracts of Futura 75, respectively 2.622% (F/10/90) and 2.682% (F/30/30). Δ^9^-THC was found to have a similar tendency as increasing ethanol concentration. In water extracts, Δ^9^-THC was detected only in hot water extract at 0.002% (F/30/30). A slightly higher but insignificant amount of Δ^9^-THC was observed in 40% extracts at the level 0.004% (F/10/90) and 0.006% (F/30/30). Significantly higher content of Δ^9^-THC at the level 0.039% (F/10/90) was found in 96% ethanol extract. However, the largest amount was found in 80% ethanol extracts, 0.045% (F/10/90) and 0.048% (F/30/30), and in 96% ethanol extract, 0.057% (F/30/30).

The variety KC Dora showed a similar tendency in CBD and Δ^9^-THC content during extraction with different methods (Table 6). The lowest and not significant amount of CBD was found in cold water extracts at the level 0.007% (K/30/90) and 0.009% (K/30/30); in hot water extracts, 0.051% (K/30/90) and 0.054% (K/30/30); and in 20% ethanol extracts from 0.064% (K/30/90) to 0.072% (K/30/30). Insignificantly higher content of CBD was observed in 40% extract at the level 0.581% (K/30/90) and 0.619% (K/30/30). Application of 80% ethanol showed a higher amount of CBD from 1.169% (K/30/90) to 1.262% (K/30/30). Extraction with 96% ethanol was the most efficient for the content of CBD, which was observed at the l 2.461% (K/30/30) and 2.468% (K/30/90). Different extraction methods also influence Δ^9^-THC content. The lowest amount (0.001%) was found in hot water extracts and 40% ethanol extract (K/30/30). The increased content at the level 0.022% was observed in 96% ethanol extract (K/30/90), 0.026% in 80% ethanol extract (K/30/90) and 0.029% in 96% ethanol extract. A significantly high content of Δ^9^-THC (0.057%) was observed in 80% ethanol extract (K/30/30).

Extraction of the variety Tygra showed the lowest content of CBD in cold water extract at the level 0.005% (T/30/30) and 0.008% (T/10/30), in hot water extracts 0.049% (T/30/30) and 0.064% (T/10/30), and in 20% ethanol extracts 0.063% (T/30/30) and 0.070% (T/10/30) (Table 7). Higher CBD content was found in 40% ethanol extracts in the amount 0.535% (T/10/30) and 0.557% (T/30/30). Application of 96% ethanol for extraction revealed the significantly highest content of CBD at the level from 1.945% (T/10/30) to 2.228% (T/30/30). The lowest content of Δ^9^-THC at the level of 0.002% was found in hot water extracts (T/10/30 and T/30/30) in 40% ethanol extract (T/10/30), and 0.003% in 40% ethanol extract (T/30/30). A higher amount of Δ^9^-THC, 0.035%, was observed in 96% ethanol extract (T/30/30) and 0.036% in 80% ethanol extract (T/30/30). The significantly highest content of Δ^9^-THC at the level of 0.045% was found in 96% ethanol extract (T/10/30) and 80% ethanol extract (T/10/30).

The use of 96% ethanol extraction with ultrasound allowed us to obtain the cannabinoid content (HPLC) (Table 4) at the level most similar to the range of cannabinoids analyzed in the plant material (GC-FID) (Table 1). Therefore, using 96% ethanol with ultrasound for extraction of cannabinoids from the plant material and HPLC analysis can be considered a suitable method for Δ^9^-THC and CBD analysis in hemp.

## 3. Discussion

Hemp cultivation requires the adjustment of nitrogen content to the local soil conditions, and in Poland optimal concentration of fertilizer is in the range of 40–120 kg⋅ha^−1^ [37,38]. The study of other authors has shown that treatment with phosphorus, nitrogen, and potassium reduces THC and CBD levels in the inflorescence leaves. Moreover, the highest content of cannabinoids occurs in the hemp upper shoots and branches and declines in older leaves [39,40]. Another author observed that differences in sowing density between the levels of 40, 80, and 120 kg⋅ha^−1^ do not significantly affect hemp yield. On the other hand, increasing the nitrogen dose and the sowing density causes slight changes in fiber yields [37].

The experiment showed no effect of nitrogen fertilization and sowing density on the content of CBD and Δ^9^-THC, suggesting that the applied doses of nitrogen were not decisive for producing cannabinoids in chosen varieties. Moreover, the obtained results showed the excellent adaptation of selected fibrous hemp varieties to the weather conditions during the study.

According to Pacifici et al., (2017), there is very little information regarding the characteristics of cannabinoids in aqueous extracts prepared from hemp [33]. The first records of cannabinoid concentration in tea obtained from narcotic cannabis indicate a reduced and heterogeneous recovery of cannabinoids, especially of pharmacologically important Δ^9^-THC. Prepared aqueous hemp extracts are a source of cannabinoids with medical potential. However, to obtain a product with a specific cannabinoid content, access to high-quality, homogeneous plant material and a stable method of cannabinoid extraction are required. Water extracts are currently not a traditional preparation method that could be used for recreational use.

The first few works on water extracts focused on the practical effects of cannabis water extracts and not on their characteristics. Nevertheless, there are mentions of the psychotropic effect of cannabis water extracts and their analgesic or non-psychotropic effect, which was due to the low content of Δ^9^-THC in cannabis tea [41,42,43,44]. At the same time, alcoholic extracts are the most common forms of preparation of pharmaceutical extracts. Therefore, the influence of temperature and concentration of the applied aqueous ethanol solution was investigated.

In their studies evaluating the ^1^H NMR spectrum of an aqueous cannabis extract, Politi et al., (2008) also recorded the absence of typical proton signals indicating the presence of Δ^9^-THC and Δ^9^-THCA, which suggests reduced extraction of psychoactive Δ^9^-THC in the aquatic environment [34]. At the same time, the absence of Δ^9^-THC in the aqueous extract obtained from the narcotic variety explains the lack of this cannabinoid in extracts obtained from typical fibrous cannabis varieties (Futura 75, KC Dora, and Tygra) with the Δ^9^-THC content in plants below 0.2%. Moreover, Politi et al., (2008) reported that the use of elevated temperature (heating and boiling for 3 min) during water extraction causes Δ^9^-THC acid decarboxylation and increases the extraction and dissolution of Δ^9^-THC in water [34]. A similar tendency was found with the use of fiber hemp. Water extraction at elevated temperature increases the Δ^9^-THC content in the extracts, related to the low Δ^9^-THC content in the plant material, but the changes were insignificant.

There is a growing interest in obtaining cannabinoid derivatives by a solvent-free method based on mechanical separation rather than extraction. The solvent-free extraction is due to the semi-fluidity of the cannabinoids, which allows them to be extracted by appropriate heating and pressure [34]. Ice water separation (“cold water extraction”) is assumed to be ideal for producing a high-quality ice hash or bubble hash. The method involves the mechanical separation of the cannabinoid-rich trichomes from the biomass using water or ice and an excitatory force. Cold-pressed marijuana or hemp oil is cooled, placed under high pressure, and crushed to press the hemp oil out of the biomass. The method maintains the desired terpenes, flavonoids, and cannabinoids, but the yield is relatively low. Cold-pressed hemp and hemp oil appear in wellness products such as tinctures and topical formulations.

As suggested by Pacifici et al., (2017), it should be considered that differences in the content of cannabinoids within the variety may result not only from the applied agrotechnical conditions but also from the heterogeneous recovery of cannabinoids [33]. Water is a poor solvent, so hemp-based water preparations are unsuitable for accurate dosing [25]. Additionally, the above theory could be confirmed by the analysis of CBD and Δ^9^-THC in plants, where agrotechnical conditions did not influence the composition of cannabinoids within the varieties. Use of water for the separation of cannabinoids needs further analysis. Recently, simple techniques to limit solvents have been revived. Hence the interest in cold water extraction is sometimes called ice water extraction or cold water extraction. Low temperatures and mixing eliminate the need for aggressive solvents, leading to a clean and solvent-free concentrate [45,46]. Coldwater extraction also produces terpenic products such as live resin and extractions with a high terpene spectrum.

Under hot, cold, or room temperature conditions, alcohol extraction is one of the most effective extraction methods for processing large batches of cannabis flowers [16,29,35]. The advantage of using ethanol extraction is its properties. The ethanol extraction process is less explosive and toxic compared to hydrocarbon extraction or supercritical extraction with CO_2_. Much cheaper infrastructure, higher bandwidth, and simplicity of use are further advantages. Due to its bipolar properties, ethanol is ideal for extracting a wide range of cannabinoids, especially at lower temperatures, and other compounds such as aromatic cannabis terpenes. During the extraction with ethanol, a crude oil is produced in the first stage, containing almost all hemp derivatives. After further refining and purification, e-cigarette oil, gel capsules, edibles, tinctures, sublingual drops, and topical products are obtained. Ethanol can also be used for large-scale extraction based on column or flash chromatography. Ethanol has its limitations. It does not purify individual cannabinoids and is not suitable for terpenes.

The obtained results from three hemp varieties demonstrated that the water extraction of cannabinoids enabled the extraction of cannabidiol without the detectable presence of Δ^9^-THC. The application of ethanol for extraction increased the level of cannabinoid extraction. The content of cannabinoids was proportional to the increasing ethanol concentration, leading to the better extraction of CBD and Δ^9^-THC. Hemp producers are increasingly starting to use ethanol extraction methods. Extraction is safe, efficient, and effective. Ethanol is often used as a food preservative and is considered “generally safe” by the FDA. The hemp plant is soaked in ethanol to extract the cannabinoids. After extraction, the product undergoes a refining process that improves the purity of the product. Ethanol will remove unwanted compounds from substances, offering one of the best-tasting and cleanest concentrates available.

Ethanol is a very efficient solvent, used in a wide range of temperatures from sub-zero up to the boiling point. Ethanol has been used for botanical extraction for thousands of years. The ethanol solvent binds to the chlorophyll, making the product taste bitter and isolating cannabinoids, terpenes, and flavonoids. Extraction tincture with alcohol allows one to obtain honey tar oil. It requires an initial stage of heating the material to about 121 °C, which leads to decarboxylation. The material is soaked in water and placed in pure, high-percentage alcohol. After draining and removing the alcohol, a classic oil is obtained.

The CBD content found in the extract at the level of 1.945% to 2.622% after applying ultrasound differs from the CBD content found in industrial hemp grown in Europe. Brighenti et al., (2017) reported the presence of CBD in selected varieties of fibrous plants at a similar level from 2.2 mg/g to 4.6 mg/g. The Futura 75 variety was also assessed, with 3.2 mg/g CBD [31].

In the case of the prepared tinctures, Politi et al., (2008) reported an increase in cannabinoid content with an increase in the concentration of ethanol used. The analysis of the ^1^H NMR spectrum in ethanol extracts prepared from Δ^9^-THC-rich cannabis and Δ^9^-THC-free varieties showed a high Δ^9^-THC content in the 80% tinctures obtained from Δ^9^-THC-rich types, but no signal corresponding to Δ^9^-THC in the Δ^9^-THC-free array [34].

The conducted research also demonstrated that the selected varieties of fibrous hemp Futura 75, KC Dora, and Tygra contained the content of Δ^9^-THC consistent with the Polish law. Glivar et al., (2020), in their research conducted in 2017–2018, found that the content of Δ^9^-THC and CBD may be affected by environmental conditions such as temperature and hydration and that a warmer and dry summer increased the range of cannabinoids [1]. The dependence of cannabinoids on environmental conditions may also explain why variations in CBD content were observed among cultivars with different requirements of seeding density and nitrogen fertilization. Other studies also indicate that using ultrasound and HPLC for the ethanol extraction of cannabinoids allows for the proper characterization of the cannabinoid profile in the plant material [1,31].

Hydrocarbon extraction based on propane, butane, or hexane has been used for many years for food extraction. Hydrocarbon extraction enables the extraction of a greater variety of terpenes from the hemp material than alcohol extraction. The preservation of the terpenes helps give the extract flavor and aroma [14,34]. The extraction of butane honey oil (BHO) is widely used in the food and perfume industries. The extraction method involves placing the cannabis in a container before spraying butane on the cannabis. Cannabinoids and butane are collected in another container. The mixture should then be allowed to evaporate, or a vacuum oven can be used to remove all butane from the variety. Extraction of BHO is dangerous due to the high flammability of the substance. THC levels when the BHO extraction method is used remain at around 80%.

Hydrocarbon extraction equipment is generally considered cheaper than CO_2_ and ethanol extraction equipment, but hydrocarbon extraction does not necessarily yield CBD and THC. Butane is mainly used on the desired product, followed by propane and hexene, due to the difference in boiling points. Mixtures of hydrocarbons may also be used. The cannabinoid concentrate obtained under the influence of liquid butane contains terpenes and cannabinoids (THC, CBD, and other smaller cannabinoids), vegetable waxes, and lipids. It is subject to further refining, including dewaxing, centrifuging, and wintering. The final step removes the hydrocarbon solvent from the extract by degassing. The use of hydrocarbons helps maintain the strain’s flavor profile, and it is a quick process using different parts of the cannabis plant. The hydrocarbon is ideal for producing edible cannabinoid derivatives such as budder, butane hash oil (BHO), crumble, honeycomb, aggregate, resin and wax, topical products, edibles, e-cigarette cartridges, tinctures, and capsules.

Edible oils such as extra virgin olive oil, coconut oil, and butter are used on a small scale to extract fat-soluble cannabinoids by gently heating the decarboxylated cannabis flowers directly in the edible oil [15]. The resulting oil is much less powerful and has a shorter shelf life than other extraction methods. In the first stage, the plant material is heated, which enables the conversion of cannabinoids into more bioavailable forms, for example, CBDA into CBD and THCA into THC by decarboxylation or decarbonization (approx. 140 °C for 30 min or 120 °C for 60 min) [15,47]. The vegetable oil is then added and heated to 100 °C for 1–2 h, allowing the decarboxylated cannabinoids to bind to the fat molecules in the oil. The plant material is filtered out, leaving a vaporized cooking oil containing a mixture of vegetable oil, terpenes, waxes, cannabinoids, etc.

CO_2_ extraction is a non-toxic, environmentally friendly method of separating cannabinoids from the cannabis plant, requiring precision equipment. It is also used in producing beer, coffee, fruit extracts, and tea. There are three main modifications: CO_2_ extraction in supercritical, subcritical, and medium-critical states [34,35]. The former is assumed to be the gold standard of extraction. However, the need for specialized equipment and skilled personnel remains an issue, leading to limitations in implementing these methods as simple and easy-to-use techniques. Due to the above, no extraction with the use of CO_2_ was undertaken in this study. Supercritical CO_2_ is heated and passed through the cannabis buds to produce waxes and oils. Low extraction temperatures at very high pressure ensure pure oil extraction. Nearly 500 cannabis compounds can be obtained [14,34,35].

Subcritical CO_2_ extraction proceeds at low temperatures and pressures, despite longer extraction times and lower yields than supercritical extraction, preserving the “full spectrum” of beneficial cannabis compounds (e.g., fine terpenes). Medium-critical CO_2_ exists in ranges of temperature and pressure between subcritical and supercritical.

Isopropyl alcohol is required for this extraction method. The dried hemp flowers are soaked in alcohol and then gently shaken. Isopropyl removes hair from the plant. The mixture is strained into a vessel, and then the solvent is removed using a vacuum oven which keeps the temperature below 181 °C. Once the solvent has evaporated, the remaining substance is oil-rich in THC. A new patent involving a new extraction method that uses algorithmic extraction to manipulate temperatures to extract cannabinoids and terpenes without losing some of the plant’s properties was prepared by Curo Medical Ltd., which specializes in the delivery of cannabis in the form of pills or sublingual strips instead of traditional smoking, vaping and eating. All extracts must be appropriately stored and refrigerated to maintain durability and quality.

## 4. Materials and Methods

### 4.1. Chemicals and Reagents

Cannabinoid reference standards for Δ^9^-THC, and CBD, were purchased from LGC Standards (Warsaw, Poland). All standards had a purity of ≥98%. N,O-bis(trimethylsilyl)trifluoroacetamide (BSTFA) was purchased from Supelco (Berlin, Germany). Acetonitrile, n-hexane for extractions, ethyl alcohol 96%, phosphoric acid, triethylamine were purchased from POCH (Warsaw, Poland). All reagents were of analytical purity.

### 4.2. Plant Material and Sampling

The hemp varieties selected for research are used in proper cultivation in Poland and the European Union. Three monoecious types of industrial hemp (*Cannabis sativa* L.) from Central Europe (KC Dora-Budapest, Hungary, Futura 75-Paris, France, and Tygra-Poznan, Poland) were grown on the fields of the Institute of Natural Fibres and Medicinal Plants in the Experimental Station in Petkowo, Wielkopolska Voivodeship (52°20’ N, 17°25’ E) in 2014. Before planting, the soil was fertilized with potassium-magnesium (300 kg⋅ha^−1^ Korn-Kali, K+S Polska, Poznan, Poland). Additional factors were different levels of nitrogen fertilization (30 and 90 kg⋅ha^−1^) and seed sowing density (10 and 30 kg of seeds/ha). The plants were collected at the late flowering phase (the beginning of seed maturation), while the inflorescences were dried on the drying floor at 40 °C for approximately 24 h. The dried material was stored in paper bags at room temperature.

### 4.3. Cannabinoids Analysis in Inflorescences

Female inflorescences have the highest content of cannabinoids. Therefore, the research focuses mainly on their assessment. Plant material samples were taken from inflorescences, then dry material was crumbled (stems and seeds were separated) and sieved through a 0.63 mm sieve. Duplicates of each sample were used for the cannabinoids analysis. According to Dussy et al., (2005), 300 mg of this fine powder was extracted with 5 mL of n-hexane for 30 min [43,48]. The extract was centrifuged and appropriately diluted in a small test tube. A 300 µL aliquot of the dilution was evaporated and heated at 130 °C for 30 min and redissolved in 600 µL of acetonitrile after BSTFA derivatization of tested analytes [49]. GC–FID analyses were performed on an AutoSystem XL chromatograph coupled to a flame ion detector (Perkin Elmer, Waltham, MA, USA). Data acquisition and analysis were performed using standard software supplied by the manufacturer (TurboChrom, PerkinElmer Corporation, Waltham, MA, USA). Substances were separated on a fused silica capillary column (SPB-5, 30 m × 0.32 mm × 0.25 µm). Temperature program: 100 °C hold for 1 min, 30° min^−1^ to 175 °C, hold for 3.5 min, 6° min^−1^ to 295 °C. The injection port and detector temperatures were 275 °C and 300 °C, respectively. Splitless injection mode was used, and helium, with a flow rate of 6.0 mL min^−1^, was used as the carrier gas. The cannabinoids were identified based on comparing their relative retention times with the corresponding values of standards assayed under the same chromatographic conditions. Quantification was performed using the standard curves. Δ^9^-tetrahydrocannabinol (Δ^9^-THC), Δ^9^-tetrahydrocannabinolic acid (Δ^9^-THCA), cannabidiol (CBD), and cannabidiol acid (CBDA) were determined.

In plants, the cannabinoids generally occur in native forms, Δ^9^-tetrahydrocannabinol acid (Δ^9^-THCA) and cannabidiolic acid (CBDA), which through the decarboxylation process become the neutral forms Δ^9^-tetrahydrocannabinol and cannabidiol. The proportions of these forms vary in plants, and the amount of neutral states increases during the storage of the material. The total content of Δ^9^-THC, Δ^9^-THCA, CBD, and CBDA remains unchanged, allowing the distinction between narcotic and fibrous varieties. Therefore the conducted research focused mainly on determining the total content of cannabinoids [31,33,50].

### 4.4. Extraction

The study indicates that temperature, solvent, and solvent polarity could influence the extraction of cannabinoids from plant material. Additionally, shaking could enhance the penetration of the solution into a cell. However, the latter effect was not observed in the experiment. Studies conducted by other groups also revealed that relative amounts of cannabinoids in the extract depend on an ethanol/water solution [51]. In the experiment, the focus was primarily on comparing extraction methods to determine the content of cannabinoids using traditional methods. The study confirms the effectiveness of extraction methods for obtaining extracts containing CBD and Δ^9^-THC from fiber hemp.

Hot water extraction was performed: 5 g of dried inflorescences were transferred to a 250 mL flask and covered with 100 mL of hot distilled water. Extraction (heating and boiling) was performed for 3 min [41]. The extract was filtered using a double-folded gauze, and the filtrate was collected.

Cold water extraction was performed as follows: 5 g of dried inflorescences were inserted into a 250 mL flask, and 100 mL of cold water was added. The flask was closed with aluminum foil and transferred to a shaker (ES 20 Shaker) for 24 h. The mixing process was performed at room temperature at a constant speed of 100 rpm [41]. After 24 h, the extract was filtered using a double-folded gauze, and the filtrate was collected.

Preparation of tinctures using 20%, 40%, and 80% ethanol was as follows: 10 g of dried inflorescences was transferred to the 250 mL flask. For maceration 100 mL of different ethanol/water mixtures (20%, 40%, and 80% *v/v*) was used. The flask was closed and covered by aluminum foil to protect against light. The samples were stirred for three days at room temperature at a speed of 100 rpm (Thermoshake Gerhardt, Königswinter, Germany), then extracts were filtered using a double-folded gauze, and the filtrates were collected [41].

Ultrasound extraction was performed as follows: 2 g of dried inflorescences were inserted into a 250 mL flask, and 10 mL of 96% ethanol was added. The samples were sonicated in an ultrasound water bath and treated with 40 kHz for 30 min [52]. After sonication, the extract was filtered using a double-folded gauze, and the filtrate was collected. Each extraction process was conducted in duplicate.

### 4.5. High-Performance Liquid Chromatography Analysis

Samples obtained from different extraction procedures were analyzed using HPLC chromatography, and separation was performed on the Accucore C18 column (2.6 μm particle size, 10 cm × 2.1 mm). Mobile phases consisted of solvents: 0.01% formic acid in acetonitrile (A) and 0.01% formic acid (B). After filtration, the samples were diluted in acetonitrile, placed in vials, and analyzed using liquid chromatography (Thermo Scientific, Waltham, MA, USA) with automatic injection (1 µL). The Accucore C18 column temperature was 50 °C. Calibration was performed by injection in triplicate of standard solutions containing CBDA, CBD, Δ^9^-THC, and Δ^9^-THCA (concentrations of 2.5 µg/mL, 5 µg/mL, and 10 µg/mL were prepared by dilution with acetonitrile). The calibration curve equation was for THC y = 0.0898x + 0.0541, where R^2^ = 0.997, limit of detection (LOD) 0.05, and for CBD y = 0.0889x + 0.0172, where R^2^ = 0.999, LOD 0.008.

The gradient was established as follows: start when 35% A, after 8 min decrease to 20% A, and 4 min later decrease to 0% A. A flow rate of 0.300 mL/min was used. The eluents were analyzed using a diode array detector (Thermo Scientific, Waltham, MA, USA) at a wavelength of 230 nm for detection. The data were collected using the software Chromeleon 7.0 (analytical error (n = 10) for Δ^9^-THCA ± 0.15%, for CBDA ± 0.10%, for CBD ± 0.5% and for Δ^9^-THC ± 0.13%, respectively). In prepared extracts, the following cannabinoids were analyzed: Δ^9^-tetrahydrocannabinol (Δ^9^-THC), Δ^9^-tetrahydrocannabinolic acid (Δ^9^-THCA), cannabidiol (CBD), and cannabidiol acid (CBDA) [25,50].

### 4.6. Statistical Analysis

Statistical analyses were performed using Statistica 12.5 software (StatSoft Polska, Cracow, Poland). The Tukey test (ANOVA, StatSoft Polska, Cracow, Poland) was carried out for each experiment, and significant differences between the content of cannabinoids were determined (α = 0.05).

## 5. Conclusions

This study aimed to compare simple methods of extracting cannabinoids from hemp. The usefulness of the selected forms for the characterization of the cannabinoid profile of hemp varieties and the potential impact of variable agrotechnical conditions were analyzed. The experiment revealed that water’s simplest extraction method was sufficient to obtain a small amount of CBD but did not allow homogeneous formulations. The investigation revealed that the simplest extraction method using water was enough to obtain a small amount of CBD but did not allow homogeneous formulations. The use of high concentrations of ethanol extracts, especially in combination with ultrasound and determination using liquid chromatography (HPLC), made it possible to obtain a cannabinoid profile corresponding to the standard cannabinoid determination using GC-FID gas chromatography. Recently, numerous modifications of traditional methods have appeared on an industrial scale, such as introducing ultrasonic aids to alcohol extraction or other types of extraction. Hydrodynamic extraction technology combines temperature, pressure, and ultrasound to create full-spectrum cannabis extracts from the whole fresh cannabis flower [16].

An attractive solution may be the use of bees fed only the full spectrum of cannabinoids. The Israeli company PhytoPharma International has produced hemp honey produced exclusively by bees. The result is natural honey infused with low concentrations of less than 0.3% active, highly bioavailable cannabinoids [53].

More methods concerning aroma preservation of bioavailability may potentially be developed using simple and easily applicable techniques.

## Figures and Tables

**Table 1:** Cannabinoids content (%) in dry hemp inflorescences from plots with different combinations of variety, sowing density (kg⋅ha^−1^) and nitrogen dose (kg⋅ha^−1^) (GC-FID method).

Variety	Plant Material	Sowing Density (kg⋅ha^−1^)	Nitrogen Dose (kg⋅ha^−1^)	Total CBD (%)	Total Δ^9^-THC (%)
Futura 75	F/10/90	10	90	2.48 ^b^	0.06 ^b^
	F/30/30	30	30	2.43 ^b^	0.05 ^b^
KC Dora	K/30/30	30	30	3.60 ^a^	0.09 ^ab^
	K/30/90	30	90	3.63 ^a^	0.10 ^ab^
Tygra	T/10/30	10	30	2.26 ^b^	0.12 ^a^
	T/30/30	30	30	2.58 ^b^	0.15 ^a^

CBD–cannabidiol, Δ^9^-THC–Δ^9^-tetrahydrocannabinol. ^a,b^ Mean values with columns with the same letter are not significantly different at α = 0.05 (Tukey’s test).

**Table 2:** Cannabinoids content (%) in the water extract prepared from the Futura 75, KC Dora, and Tygra varieties (HPLC method).

Extraction Method	Variety	Plant Material	Total CBD (%)	Total Δ^9^-THC (%)
Cold water	Futura 75	F/10/90	0.007 ± 0.000 ^a^	0.000 ± 0.000
		F/30/30	0.007 ± 0.002 ^a^	0.000 ± 0.000
	KC Dora	K/30/30	0.009 ± 0.002 ^a^	0.000 ± 0.000
		K/30/90	0.007 ± 0.000 ^a^	0.000 ± 0.000
	Tygra	T/10/30	0.008 ± 0.001 ^a^	0.000 ± 0.000
		T/30/30	0.005 ± 0.002 ^a^	0.000 ± 0.000
Hot water	Futura 75	F/10/90	0.063 ± 0.006 ^c^	0.000 ± 0.000
		F/30/30	0.048 ± 0.004 ^b^	0.002 ± 0.000
	KC Dora	K/30/30	0.054 ± 0.002 ^bc^	0.001 ± 0.000
		K/30/90	0.051 ± 0.003 ^b^	0.001 ± 0.000
	Tygra	T/10/30	0.064 ± 0.003 ^c^	0.002 ± 0.000
		T/30/30	0.049 ± 0.001 ^b^	0.002 ± 0.000

CBD–cannabidiol, Δ^9^-THC–Δ^9^-tetrahydrocannabinol. ^a–c^ Mean values in a column with the same letter are not significantly different at α = 0.05 (Tukey’s test).

**Table 3:** Cannabinoids content (%) in tinctures (20% ethanol, 40% ethanol, and 80% ethanol) prepared from inflorescences of the Futura 75, KC Dora, and Tygra varieties (HPLC method).

Extraction Method (100 rpm, 72 h)	Variety	Plant Material	Total CBD (%)	Total Δ^9^-THC (%)
20% EtOH	Futura 75	F/10/90	0.081 ± 0.009 ^a^	0.000 ± 0.000 ^a^
		F/30/30	0.071 ± 0.005 ^a^	0.000 ± 0.000 ^a^
	KC Dora	K/30/30	0.072 ± 0.005 ^a^	0.000 ± 0.000 ^a^
		K/30/90	0.064 ± 0.004 ^a^	0.000 ± 0.000 ^a^
	Tygra	T/10/30	0.070 ± 0.006 ^a^	0.000 ± 0.000 ^a^
		T/30/30	0.063 ± 0.011 ^a^	0.000 ± 0.000 ^a^
40% EtOH	Futura 75	F/10/90	0.581 ± 0.093 ^b^	0.004 ± 0.003 ^a^
		F/30/30	0.616 ± 0.041 ^b^	0.006 ± 0.002 ^ab^
	KC Dora	K/30/30	0.619 ± 0.021 ^b^	0.001 ± 0.000 ^a^
		K/30/90	0.581 ± 0.005 ^b^	0.000 ± 0.000 ^a^
	Tygra	T/10/30	0.535 ± 0.037 ^b^	0.002 ± 0.000 ^a^
		T/30/30	0.557 ± 0.021 ^b^	0.003 ± 0.001 ^a^
80% EtOH	Futura 75	F/10/90	1.305 ± 0.000 ^ef^	0.045 ± 0.020 ^bc^
		F/30/30	1.393 ± 0.005 ^f^	0.048 ± 0.026 ^c^
	KC Dora	K/30/30	1.262 ± 0.037 ^ef^	0.057 ± 0.002 ^c^
		K/30/90	1.169 ± 0.022 ^de^	0.026 ± 0.000 ^abc^
	Tygra	T/10/30	0.997 ± 0.040 ^cd^	0.045 ± 0.007 ^bc^
		T/30/30	0.952 ± 0.160 ^c^	0.036 ± 0.025 ^abc^

CBD–cannabidiol, Δ^9^-THC–Δ^9^-tetrahydrocannabinol. ^a–f^ Mean values with a column with the same letter are not significantly different at α = 0.05 (Tukey’s test).

**Table 4:** Cannabinoids content (%) in 96% ethanol extracts prepared from the Futura 75, KC Dora, and Tygra varieties (HPLC method).

Extraction Method (45 Hz, 0.5 h)	Variety	Plant Material	Total CBD (%)	Total Δ^9^-THC (%)
96% EtOH	Futura 75	F/10/90	2.622 ± 0.141	0.039 ± 0.001
		F/30/30	2.682 ± 0.314	0.057 ± 0.011
	KC Dora	K/30/30	2.461 ± 0.009	0.029 ± 0.001
		K/30/90	2.468 ± 0.598	0.022 ± 0.001
	Tygra	T/10/30	1.945 ± 0.137	0.045 ± 0.021
		T/30/30	2.228 ± 0.246	0.035 ± 0.010

CBD–cannabidiol, Δ^9^-THC–Δ^9^-tetrahydrocannabinol.

**Table 5:** Cannabinoids content (%) in water and ethanol extracts prepared from the Futura 75 variety (HPLC method).

Extraction Method	Plant Material	Total CBD (%)	Total Δ^9^-THC (%)
Cold water	F/10/90	0.007 ± 0.000 ^a^	0.000 ± 0.000 ^a^
	F/30/30	0.007 ± 0.002 ^a^	0.000 ± 0.000 ^a^
Hot water	F/10/90	0.063 ± 0.006 ^a^	0.000 ± 0.000 ^a^
	F/30/30	0.048 ± 0.004 ^a^	0.002 ± 0.000 ^a^
20% EtOH	F/10/90	0.081 ± 0.009 ^a^	0.000 ± 0.000 ^a^
	F/30/30	0.071 ± 0.005 ^a^	0.000 ± 0.000 ^a^
40% EtOH	F/10/90	0.581 ± 0.093 ^a^	0.004 ± 0.003 ^a^
	F/30/30	0.616 ± 0.041 ^a^	0.006 ± 0.002 ^a^
80% EtOH	F/10/90	1.305 ± 0.000 ^b^	0.045 ± 0.020 ^b^
	F/30/30	1.393 ± 0.005 ^b^	0.048 ± 0.026 ^b^
96% EtOH	F/10/90	2.622 ± 0.141 ^c^	0.039 ± 0.001 ^ab^
	F/30/30	2.682 ± 0.314 ^c^	0.057 ± 0.011 ^b^

CBD–cannabidiol, Δ^9^-THC–Δ^9^-tetrahydrocannabinol. ^a–c^ Mean values in column with the same letter are not significantly different at α = 0.05 (Tukey’s test).

**Table 6:** Cannabinoids content (%) in water and ethanol extracts prepared from the KC Dora variety (HPLC method).

Extraction Method	Plant Material	Total CBD (%)	Total Δ^9^-THC (%)
Cold water	K/30/30	0.009 ± 0.002 ^a^	0.000 ± 0.000 ^a^
	K/30/90	0.007 ± 0.000 ^a^	0.000 ± 0.000 ^a^
Hot water	K/30/30	0.054 ± 0.002 ^a^	0.001 ± 0.000 ^a^
	K/30/90	0.051 ± 0.003 ^a^	0.001 ± 0.000 ^a^
20% EtOH	K/30/30	0.072 ± 0.005 ^a^	0.000 ± 0.000 ^a^
	K/30/90	0.064 ± 0.004 ^a^	0.000 ± 0.000 ^a^
40% EtOH	K/30/30	0.619 ± 0.021 ^ab^	0.001 ± 0.000 ^a^
	K/30/90	0.581 ± 0.005 ^ab^	0.000 ± 0.000 ^a^
80% EtOH	K/30/30	1.262 ± 0.037 ^b^	0.057 ± 0.002 ^d^
	K/30/90	1.169 ± 0.022 ^b^	0.026 ± 0.000 ^bc^
96% EtOH	K/30/30	2.461 ± 0.009 ^c^	0.029 ± 0.001 ^c^
	K/30/90	2.468 ± 0.598 ^c^	0.022 ± 0.001 ^b^

CBD–cannabidiol, Δ^9^-THC–Δ^9^-tetrahydrocannabinol. ^a–c^ Mean values in column with the same letter are not significantly different at α = 0.05 (Tukey’s test).

**Table 7:** Cannabinoids content (%) in water and ethanol extracts prepared from the Tygra variety (HPLC method).

Extraction Method	Plant Material	Total CBD (%)	Total Δ^9^-THC (%)
Cold water	T/10/30	0.008 ± 0.001 ^a^	0.000 ± 0.000 ^a^
	T/30/30	0.005 ± 0.002 ^a^	0.000 ± 0.000 ^a^
Hot water	T/10/30	0.064 ± 0.003 ^a^	0.002 ± 0.000 ^a^
	T/30/30	0.049 ± 0.001 ^a^	0.002 ± 0.000 ^a^
20% EtOH	T/10/30	0.070 ± 0.006 ^a^	0.000 ± 0.000 ^a^
	T/30/30	0.063 ± 0.011 ^a^	0.000 ± 0.000 ^a^
40% EtOH	T/10/30	0.535 ± 0.037 ^b^	0.002 ± 0.000 ^a^
	T/30/30	0.557 ± 0.021 ^b^	0.003 ± 0.001 ^a^
80% EtOH	T/10/30	0.997 ± 0.040 ^c^	0.045 ± 0.007 ^b^
	T/30/30	0.952 ± 0.160 ^c^	0.036 ± 0.025 ^ab^
96% EtOH	T/10/30	1.945 ± 0.137 ^d^	0.045 ± 0.021 ^b^
	T/30/30	2.228 ± 0.246 ^d^	0.035 ± 0.010 ^ab^

CBD–cannabidiol, Δ^9^-THC–Δ^9^-tetrahydrocannabinol. ^a–d^ Mean values in column with the same letter are not significantly different at α = 0.05 (Tukey’s test).

## Data Availability

Data sharing is not applicable.

## Sample Availability

Not available.
